# Study on the Flexural Deformation Behavior of High-Titanium Heavy-Slag Concrete Composite Beams: Material Application, Experimental Investigation, and Theoretical Refinement

**DOI:** 10.3390/ma17194721

**Published:** 2024-09-26

**Authors:** Jinkun Sun, Yun Yu, Rita Yi Man Li, Zilin Wang, Lindong Li, Feifei Guo, Liangliang Yu, Chenxi Deng

**Affiliations:** 1 School of Civil and Architectural Engineering, Panzhihua University, Panzhihua 617000, China; 19102845524@163.com (Z.W.); lilindong@pzhu.edu.cn (L.L.); seawater0807@163.com (F.G.); shushengbeibei@163.com (L.Y.); dcx779611715@163.com (C.D.); 2 Rattanakosin International College of Creative Entrepreneurship, Rajamangala University of Technology Rattanakosin, Bangkok 10700, Thailand; 3 Panzhihua Gangcheng Group Co., Ltd., Panzhihua 617099, China; yuyun-ss@163.com; 4 Sustainable Real Estate Research Center, Hong Kong Shue Yan University, Hong Kong 999077, China; ymli@hksyu.edu

**Keywords:** high-titanium heavy-slag concrete, composite beams, deflection, cracks, correlation analysis

## Abstract

To investigate the flexural performance of high-titanium heavy-slag concrete composite beams under loading, this study examined the impact of various factors on deflection development and crack propagation as well as the applicability of empirical formulas for monolithic concrete beams. Seven concrete beams were fabricated with variables such as the reinforcement ratio, prefabrication height, and material composition, and were subjected to two-point concentrated loading. By comparing deflection values and crack widths during loading and analyzing the correlations with empirical formulas from standards, theoretical formulas with significant deviations were modified and compared. The study indicated that the cracking moment and deflection correlated with the reinforcement ratio, material structure combination, and composite height. The empirical formulas for the maximum crack width and deflection of flexural members were applicable to high-titanium heavy-slag concrete composite beams, although some discrepancies existed compared with the experimental values. After modifications, these discrepancies were reduced. This research provides a comprehensive analysis of the deformation characteristics and fracture behavior of high-titanium heavy-slag concrete composite beams.

## 1. Introduction

As global environmental issues become increasingly prominent, the sustainable development of engineering construction activities is receiving growing attention [1,2,3]. The excessive extraction of construction materials such as sand and gravel has led to a severe shortage of these resources. Simultaneously, production activities generate vast amounts of construction waste [4,5], occupying extensive land resources and imposing a significant burden on the environment [6,7,8].

After smelting magnetite, the Panzhihua Iron and Steel Group produces large quantities of high-titanium heavy slag, an industrial solid waste [9]. Current technology struggles to effectively refine this slag, resulting in its prolonged accumulation and occupation of land resources. Researchers have found that high-titanium heavy slag is a good alternative material for concrete aggregates, possessing significant recycling and reuse values as well as unique advantages [10,11,12]. Sun J et al. [13] determined the mix ratio of high-titanium heavy-slag concrete using orthogonal experiments, verifying the mechanical properties of lightweight composite slabs made from this concrete. Zhang T et al. [14] found that high-titanium heavy slag can promote the hydration reaction of cement, reduce concrete porosity, and increase interfacial strength. Lin-Ze LI et al. [15] explored the impact of high-titanium heavy-slag incorporation on the carbonation depth of concrete, proposing related functional relationships. Zhong [16] confirmed the excellent frost resistance of high-titanium heavy-slag concrete. These studies have explored the properties of high-titanium heavy-slag concrete from various perspectives, partially revealing its material characteristics when used in different structures.

Prefabricated construction, which involves factory production, transportation to the site, and on-site assembly, is a new building model with advantages such as rapid construction, a high engineering quality, reduced labor consumption, and environmental sustainability [17,18,19]. Composite concrete beams, commonly used in prefabricated buildings [20,21], offer high construction efficiency and a low environmental impact, making them widely utilized in practical engineering [22]. Du [23] studied the bending performance of glued laminated timber–concrete composite beams and proposed an optimization scheme for the load-bearing capacity of these beams. Ranjbar, Navid [24] investigated the bending failure mode, ultimate load, and deflection characteristics of geopolymer concrete beams. Gohnert [25] examined the horizontal shear performance of composite-beam interfaces, analyzing the relationship between concrete surface roughness and compressive strength. These studies have partially revealed the impact of different material properties on the mechanical performance of concrete composite beams.

The deflection and cracking of concrete beams are closely related to their safety performance, garnering significant attention from scholars [26,27]. Variations in material selection and structural composition can result in differences in the crack resistance of concrete beams, prompting extensive research into structural reinforcement and optimal material design. Khorasani [28] studied the relationship between the reinforcement ratio and the development of deflection and cracking in concrete beams. Chaudhary [29] developed a model to predict the inelastic mid-span deflection and elastic mid-span deflection of continuous composite beams. Zou [30] discussed experimental results on the deflection and cracking of carbon-fiber-reinforced polymer (CFRP) prestressed concrete beams under sustained long-term loading. Andreaus [31] proposed a new method for damage detection in multi-cracked beam structures based on a static deflection analysis. Gouda [32] investigated the effects of glass-fiber-reinforced polymer (GFRP) on crack propagation, deflection behavior, and the variation in kb values under different crack widths in concrete. Pansare [33] demonstrated the effectiveness of the static deflection method when detecting diagonal cracks in cantilever beams. Nie [34] developed a time-dependent analysis model to predict the long-term deflection of cracked reinforced concrete beams under sustained loading. Mazaheripour [35] studied the combination of a non-corrosive, low-modulus GFRP with prestressed steel strands, achieving a favorable balance in reinforcement effectiveness, ductility, durability, and economic cost. Through experimental investigation, theoretical analysis, and model prediction, the development of deflection and cracking in concrete beams has been partially revealed.

In summary, utilizing high-titanium slag as an aggregate in concrete can alleviate the global shortage of sand and gravel resources while effectively addressing the issue of industrial solid waste occupying land resources. Although scholars have studied the deformation performance of components made from high-titanium heavy slag, composite beams with different materials, and flexural concrete members, the use of high-titanium heavy-slag concrete in composite beams remains limited. The following issues urgently require clarification. 1. The relationship between the fracture characteristics and deflection development of flexural composite beams made from high-titanium heavy-slag concrete and variables such as the reinforcement ratio, prefabricated height of composite beams, and material structure composition remains unclear. 2. There are currently no established standards or regulations regarding the cracking and deformation of flexural members made from high-titanium heavy-slag concrete. To better utilize and promote high-titanium heavy slag in composite-beam construction, it is essential to conduct a comparative analysis of the factors causing crack development and deformation deflection in composite beams. Clarifying the relationship between these variables and the deformation characteristics of composite beams will help to improve the relevant theoretical framework.

Based on this, our study tracks the crack development and deflection progression of seven concrete composite beams under flexural loading using the reinforcement ratio, prefabricated height, and composite type as variables. For comparison, the study establishes a correlation between the material properties and deformation performance, comparing the results with those obtained from standardized empirical formulas. Finally, relevant coefficients are introduced through linear fitting to modify significantly divergent empirical formulas. This research provides a comprehensive exploration of the flexural performance of high-titanium heavy-slag concrete composite beams, offering a theoretical foundation and technical support for their application in composite-beam construction.

## 2. Preparation of Raw Materials and Samples

### 2.1. Materials

The raw materials used in the study included cement, coarse aggregates, fine aggregates, a water reducer, fly ash, silica fume, water, and steel reinforcements. The basic characteristics of each material are described below.

#### 2.1.1. Cement

Cement, as the most commonly used binding material in concrete, directly influences the mechanical properties of concrete. This study used ordinary Portland cement (PO42.5), produced by Yunnan Yimen Dachunshu Cement Co., Ltd. (Yuxi, China). Its characteristics are detailed in Table 1.

#### 2.1.2. Coarse Aggregates

**Figure 1:**
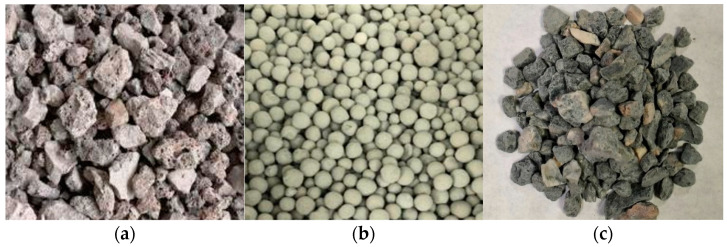
Coarse aggregates: (**a**) high-titanium heavy slag; (**b**) fly-ash lightweight aggregates; (**c**) limestone.

The coarse aggregates selected for the study included high-titanium heavy-slag crushed stone, lightweight aggregates made from fly ash, and limestone. The high-titanium heavy-slag crushed stone, produced by Panzhihua Iron and Steel Group (Panzhihua, China), had a particle size ranging from 2 mm to 26 mm, an apparent density of 2840 kg/m^3^, and a bulk density of 1350 kg/m^3^. Fly-ash lightweight aggregates, characterized by a low cost, excellent performance, low thermal conductivity, and strong thermal stability [36], were used. The high-titanium heavy slag contained partially refined titanium metal, resulting in a higher density. To reduce the weight of the high-titanium heavy-slag concrete composite beams [37] and improve their flexural performance, fly-ash lightweight aggregates were added to the concrete, based on the existing literature and research experience. The particle size of the fly-ash lightweight aggregates ranged from 2 mm to 20 mm. The grading of the high-titanium heavy-slag crushed stone is shown in Table 2, and was obtained by using square-hole sieves, as depicted in Figure 1.

#### 2.1.3. Fine Aggregates

The study utilized high-titanium heavy-slag sand and limestone fines as fine aggregates, which were sourced from Panzhihua Iron and Steel Group. The fineness modulus *Mx* was 2.9, with an apparent density of 3140 kg/m^3^ and a bulk density of 1680 kg/m^3^.

#### 2.1.4. Steel Bars

The study used ordinary hot-rolled ribbed steel bars of HRB400 grade for reinforcement. The stirrups used C steel bars, the ties used C10 steel bars, and the longitudinal bars used C16, C18, and C20 steel bars. Two sets of steel bars with different diameters were reserved for testing under the same conditions; each set was 550 mm long. To obtain accurate tensile strength data for the steel bars before beam testing commenced, tensile strength tests were conducted, as shown in Table 3.

### 2.2. Test Specimen Preparation

#### 2.2.1. Flexural Member Design

(1) Concrete Mix Design

To investigate the flexural performance of high-titanium heavy-slag concrete composite beams and explore the effects of factors such as the reinforcement ratio, precast layer height, and composite-beam type on the flexural performance of composite beams in positive bending sections, two different materials were designed for this experiment. These were ordinary concrete and high-titanium heavy-slag concrete. In the subsequent experiments, ordinary concrete was used in the structure of the ordinary concrete composite beams, while high-titanium slag concrete was utilized in the structure of the high-titanium slag composite beams. The mix designs for both materials are shown in Table 4 and Table 5.

(2) Reinforcement Layout

**Figure 2:**
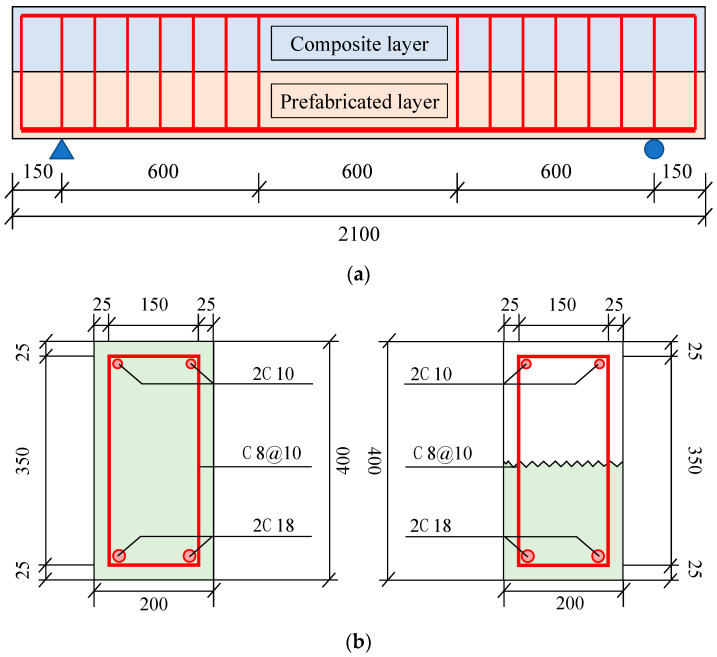
Reinforcement layout diagram (unit: mm): (**a**) Main view, (**b**) Left view.

The composite beam had a span of 2.1 m, with cross-sectional dimensions of 200 mm by 400 mm and a concrete cover thickness of 25 mm. To ensure optimal interface bonding between the upper and lower parts of the composite beam, the top surface of the precast layer was roughened. The tensile reinforcement consisted of 2C16 (HRB400), 2C18 (HRB400), and 2C20 (HRB400) bars. All supporting bars were 2C10 (HRB400). Stirrups were arranged at C8@10 mm in the region outside the pure bending section and within the support area. The reinforcement distribution is illustrated in Figure 2.

To investigate the flexural performance of high-titanium heavy-slag concrete composite beams and to explore the effects of the reinforcement ratio, prefabricated layer height, and composite-beam types on the flexural performance of the composite beam’s normal section, this experiment designed seven flexural test beams. Among them, one was a monolithically cast high-titanium heavy-slag comparison beam (HTC beam), five were high-titanium heavy-slag composite beams (HTC composite beams), and one was a normal concrete composite beam (OPC composite beam). The prefabricated heights of the composite beams were set at 150 mm, 200 mm, and 250 mm. Specific information and casting schemes for the specimens are detailed in Table 6.

#### 2.2.2. Specimen Fabrication and Measuring-Point Arrangement

The specimens were cast using wooden molds. First, the steel bars were tied in place and spacers were placed in the molds to ensure the concrete cover thickness met the design requirements. The steel-bar cage was then placed into the molds and secured with wire to prevent deformation during casting. Concrete was poured in two stages; the second pour occurred 14 days after the first. The top surface of the prefabricated layer was roughened after the first pour. For each pour, one set of high-titanium heavy-slag concrete specimens, one set of ordinary concrete specimens, and a concrete temperature compensation specimen were reserved. Each set comprised three blocks. The molds were removed 24 h after casting, and the specimens were cured by covering them with a layer of burlap and daily watering for 28 days. Before the flexural tests, the specimens were whitewashed and marked with a 50 × 50 mm grid to facilitate the observation of crack development.

**Figure 3:**
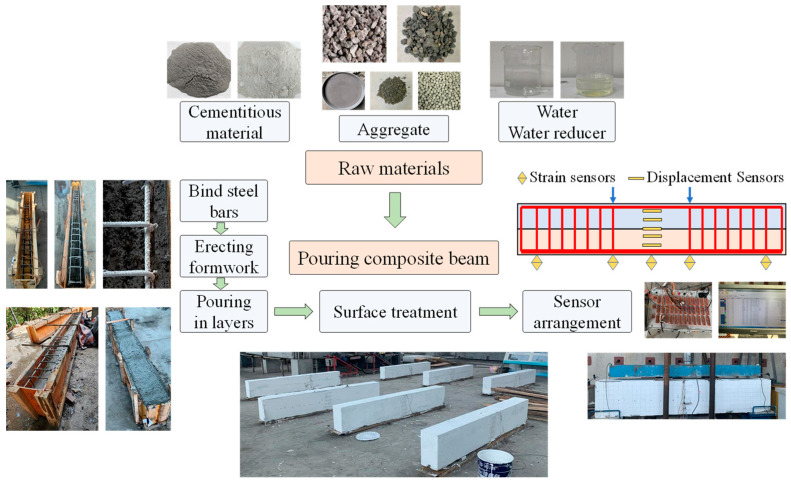
Fabrication of concrete composite beams and sensor layout.

To investigate the crack development and deflection behavior of the reinforced concrete beams during loading, strain gauges and displacement meters were used to monitor the process [38,39]. Six strain gauges were placed at the mid-span of the concrete beam, with five located on the sides of the beam and one on the top. Five displacement meters were installed at the mid-span, the loading points, and both support ends. The arrangement of the sensors on the specimens is shown in Figure 3.

## 3. Experimental Design

To investigate the damage process of high-titanium slag concrete composite beams under bending loads, hydraulic jacks were used for two-point concentrated loading through a distribution beam. Sensors monitored the load values in real-time. Fine sand was used to level the supports as well as the simply supported points of the distribution beam before loading.

Before the experiment, the components were preloaded to check the functionality of the loading equipment and other instruments. After preloading, the formal loading commenced. Until the specimen cracked, loading was conducted at a rate of 10 kN/min. Upon reaching 50 kN, the rate was reduced to 5 kN/min. Once the specimen cracked, the loading rate was restored to 10 kN/min. When the load reached 90% of the bending capacity, the rate was again reduced to 5 kN/min.

**Figure 4:**
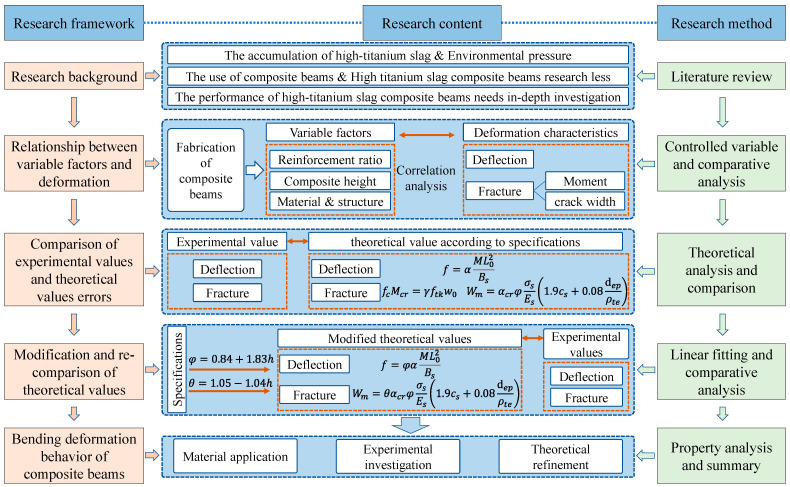
Flowchart of the research procedure.

We observed the deflection development and crack propagation process of composite beams under different variables to explore the factors affecting beam deformation. We compared the experimental values with the theoretical values calculated by empirical formulas provided in the standards, and adjusted the formulas with significant deviations. Finally, we compared the modified theoretical formulas with the experimental values. The research methodology is illustrated in Figure 4.

## 4. Results and Discussion

To investigate the relationship between different variables and the performance of composite concrete beams, the beams were grouped based on various variables. Beams L1, L2, and L3 had different reinforcement diameters to explore the impact of the reinforcement ratio on the composite-beam performance. Beams L2, L4, L5, and L6 were used to study the effect of the composite height on the beam performance. Beams L4, L6, and L7 were used to examine the influence of material structure combinations on the beam performance.

### 4.1. Test Results and Analysis of Deflection Development in Concrete Beams

**Figure 5:**
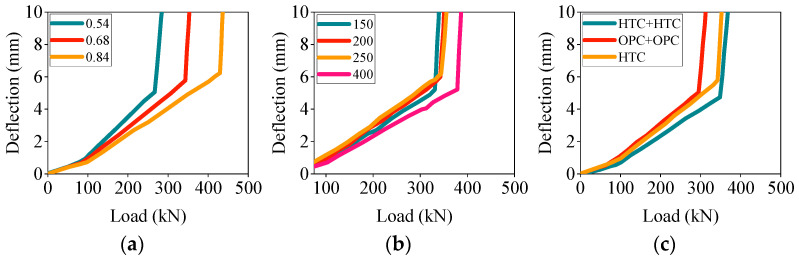
Deflection–load curve: (**a**) reinforcement ratio; (**b**) composite height; (**c**) material structure combination.

This experiment investigated the effect of different variables on the deflection development of high-titanium heavy-slag concrete composite beams. The relationship between various variables and the deflection development of the concrete composite beams is shown in Figure 5.

As shown in Figure 5a, when other variables were constant, the deflection of the concrete composite beams with different reinforcement ratios significantly varied. Specifically, at the same load, the concrete composite beam with a reinforcement ratio of 0.84 exhibited smaller deflection, while the beam with a reinforcement ratio of 0.54 showed greater deflection. This indicated that increasing the reinforcement ratio within a certain range could reduce the deflection of high-titanium heavy-slag concrete composite beams under loading. This was because the tensile strength of the steel reinforcement far exceeded that of the concrete and an increased reinforcement ratio enlarged the contact area between the steel and concrete, significantly enhancing the concrete, thereby effectively reducing the deformation of the concrete beam under loading. Additionally, variations in the prefabricated height also affected the deflection of the concrete composite beams, warranting a further in-depth analysis. Compared with monolithic casting, the composite beams exhibited a smaller deflection under the same load, with the high-titanium heavy-slag concrete composite beams showing the least deflection. Although high-titanium heavy slag has a relatively high density, the incorporation of fly-ash ceramsite reduces the composite beam’s weight. Both materials possess excellent properties that effectively decrease the deflection of flexural members. The mechanisms behind the improved deformation performance of the high-titanium heavy-slag concrete composite beams require a further detailed analysis and an explanation.

In summary, to reduce the deflection of concrete beams, it is advisable to appropriately increase the reinforcement ratio and use the material structure combination of high-titanium heavy-slag composite beams.

### 4.2. Comparison and Correction of Empirical Formulas for Mid-Span Deflection

#### 4.2.1. Comparison of Empirical Formulas for Mid-Span Deflection

To further explore the relationship between deflection development in composite beams and various variables, experimental values were compared with theoretical values. The theoretical range for the calculation of beam deflection applies from the cracking of the beam component until the yielding of the longitudinal reinforcement. According to relevant standards [40], the deflection calculation formulas for flexural members are as follows:

(1) $$f=\alpha \frac{ML_{0}^{2}}{B_{s}}$$

(2) $$B_{s}=\frac{E_{s}A_{s}h_{0}^{2}}{1.15\varphi +0.2+\frac{6\alpha_{E}\rho }{1+3.5\gamma_{f}^{\prime }}}$$

where M is the bending moment borne by the beam’s span, $L_{0}$ is the length of the beam’s span, $B_{s}$ is the short-term stiffness of the flexural member, φ is the coefficient of the non-uniform strain of reinforcement, $\alpha_{E}$ is the ratio of the elastic modulus of the steel reinforcement to the elastic modulus of the concrete, ρ is the longitudinal reinforcement ratio of the tensile steel reinforcement, $\gamma_{f}^{\prime }$ is the ratio of the tensile flange section area to the effective section area of the web, $E_{s}$ is the modulus of elasticity of the reinforcing steel, $A_{s}$ is the cross-sectional area of the tensioned reinforcing steel, and $h_{0}$ is the effective depth of the beam’s cross-section.

**Figure 6:**
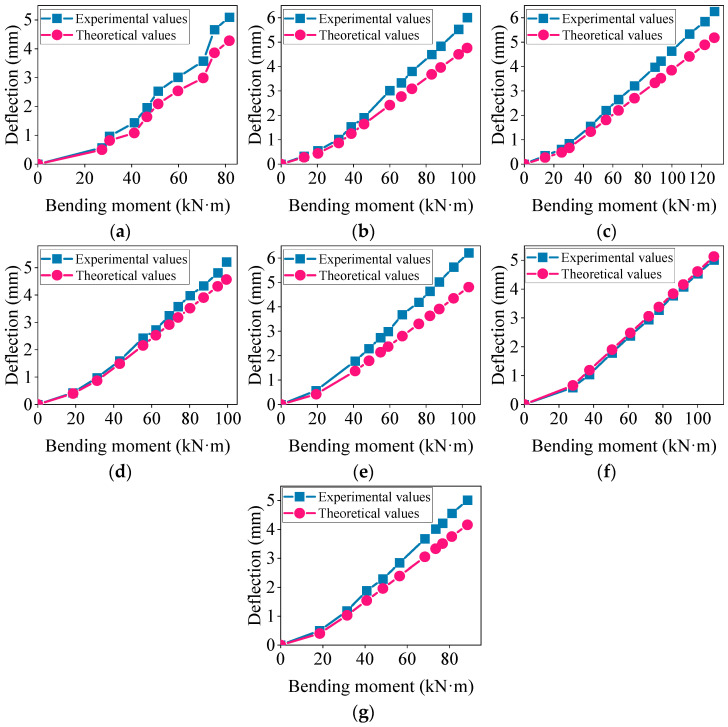
Comparison of experimental values and standard theoretical values for short-term deflection: (**a**–**g**) represent the composite beams L1~L7.

Based on empirical formulas and experimental results, the comparison chart of the short-term deflection experimental values and the standard theoretical values is shown in Figure 6. Panels (a) to (g) represent the deflection development of concrete beams L1 to L7, respectively.

As shown in Figure 6, the experimental mid-span deflection values for the L6 high-titanium heavy-slag monolithic beam closely matched the standard theoretical values. However, for the other composite beams, there were significant discrepancies between the experimental and theoretical deflection values. Under the same load, the experimental deflection values of the composite beams were greater than the theoretical values, and this difference increased with the load. This suggests that the actual deflection of the composite beams under loading may have exceed the theoretically designed values, potentially negating the safety margin considered during the design [41].

#### 4.2.2. Correction and Re-Comparison of Empirical Formulas for Mid-Span Deflection

To improve the accuracy of the deflection calculation formulas for composite beams and improve the precision of the theoretical system, the theoretical and experimental deflection values of the composite beams were linearly regressed based on the prefabricated height. A correction coefficient *φ* was introduced to the linear regression equation as follows [40]:

(3) φ=0.84+1.83h

(4) $$f=\varphi \alpha \frac{ML_{0}^{2}}{B_{s}}$$

where h represents the prefabricated height of the composite beam in meters. The definitions of the other parameters were the same as those listed in Section 4.2.1.

**Figure 7:**
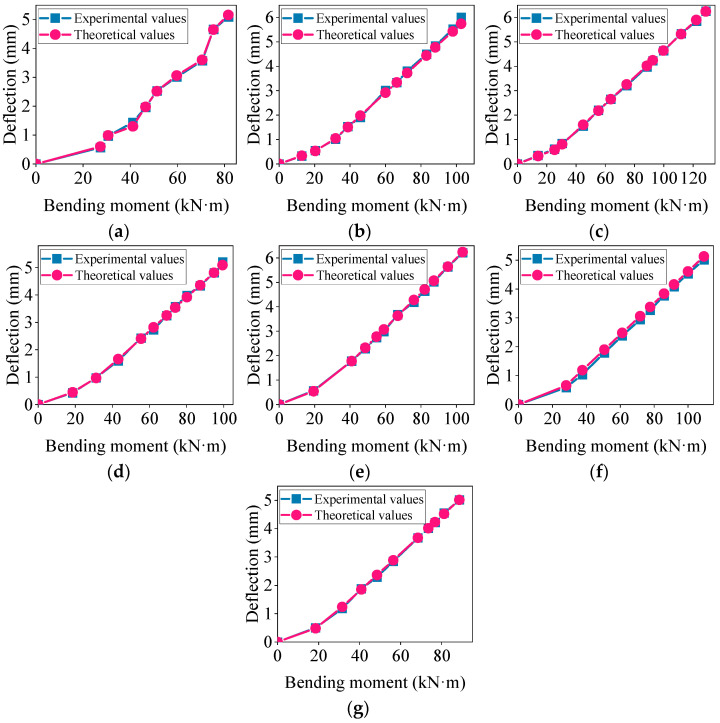
Comparison of experimental values and modified theoretical values for short-term deflection: (**a**–**g**) represent the composite beams L1~L7.

Using the modified theoretical formulas, the deflection of the concrete beams was calculated and the experimental values were compared with the modified theoretical values. The results are shown in Figure 7.

**Figure 8:**
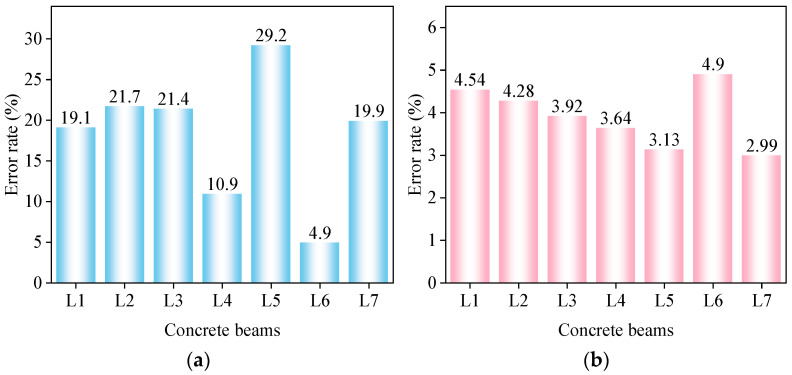
Error comparison of deflection: (**a**) standard theoretical values; (**b**) modified theoretical values.

Figure 7 illustrates that by introducing a modification factor *φ* for the height of the prefabricated part of the composite beam, the modified theoretical short-term deflection values of the concrete composite beam aligned more closely with the experimental values, resulting in a smaller deviation in the load–deflection curve. To further quantify the error between the modified theoretical values and the standard theoretical values, a calculation formula for the deflection error was introduced, as shown below [36].

(5) $$E=\frac{\left|\sum_{i=1}^{n}{f_{tei}-f}_{thi}\right|}{\sum_{i=1}^{n}f_{tei}}\times 100\%$$

where E is the calculation error, $f_{tei}$ is the deflection of the concrete beam obtained after experimental testing, and $f_{thi}$ is the deflection of the concrete beam derived from the formula. The error comparison between the modified theoretical deflection values and the standard theoretical values of the concrete composite beam according to the calculations is shown in Figure 8.

Figure 8 shows that before modification, the deviation between the standard theoretical values and the modified theoretical values of the concrete composite beam was significant, with L4 having a deviation of 10.9%, L5 a deviation of 29.2%, and the remaining composite beams around 20%. After introducing the modification factor, the deviation between the theoretical calculation values and the experimental values of the composite beam was within 5%, demonstrating that the modification of the theoretical formula significantly improved the accuracy.

### 4.3. Test Results and Analysis of Concrete-Beam Crack Development

The experiment investigated the effects of different variables on the deflection development of high-titanium heavy-slag concrete composite beams. Based on the load value F obtained from the experiment, the moment M experienced by the specimen could be calculated as follows [40]:

(6) $$M=\frac{1}{2}Fl$$

where *l* represents the distance between the loading point at the top of the beam and the support point at the bottom. In this study, l = 0.6 m.

**Figure 9:**
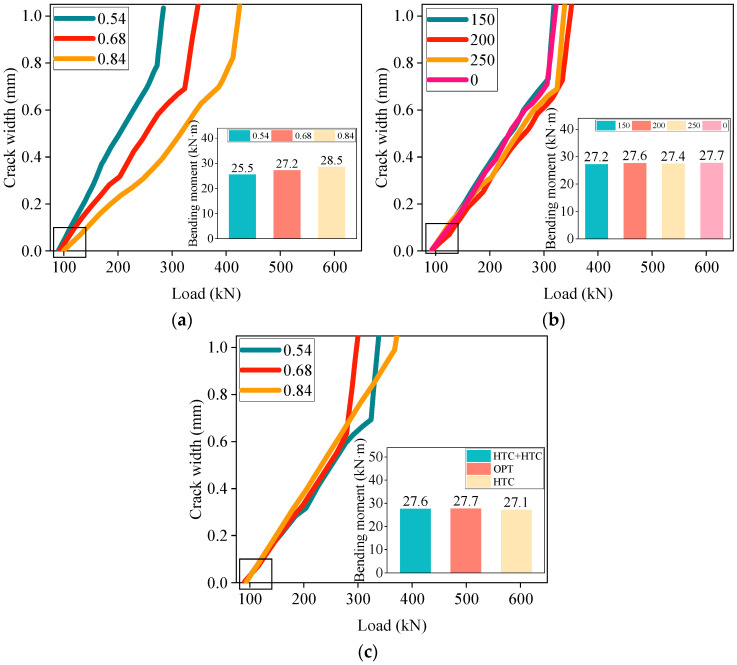
Crack development: (**a**) reinforcement ratio; (**b**) composite height; (**c**) material structure combination.

The relationship between various variables and crack development in the concrete composite beams is illustrated in Figure 9.

Through experimentation, it was observed that the crack development in seven different beams was similar. The initial cracks all originated near the point of the pure bending moment, accompanied by a slight noise. As the load increased, the crack width widened and new cracks continued to appear both in the bending–shear zones and pure bending zones, propagating towards the loading point.

Figure 9a shows that under the same load, an increase in the reinforcement ratio appropriately reduced the maximum crack width in the beam, indicating that higher reinforcement ratios could moderately suppress crack development. Additionally, when the reinforcement ratios were 0.54, 0.68, and 0.84, the cracking moments $w_{0}$ were 25.5 kN·m, 27.2 kN·m, and 28.5 kN·m, respectively. This demonstrated that increasing the reinforcement ratio could somewhat inhibit crack development. This was because the reinforcement could replace the tensile role of the concrete, resulting in smaller deformations of the concrete beam under the same load and reducing the likelihood of cracking. Figure 9b shows that appropriately increasing the prefabricated height resulted in an increase in the crack width, with the cracking moments slightly varying for different prefabricated heights. Furthermore, Figure 9c reveals that the crack widths under the same load differed among the three composite beams. Overall, the crack width of the high-titanium heavy-slag composite beam was smaller, while the crack width of the ordinary concrete composite beam was larger. This demonstrated that the high-titanium heavy slag and fly-ash ceramsite exhibited excellent properties in the flexural members, with both materials enhancing the composite beam’s crack-resistance performance.

The experiment investigated the effects of different variables on the flexural cracks in high-titanium heavy-slag concrete composite beams. Our overall analysis suggests that it is advisable to appropriately increase the reinforcement ratio and utilize a material structure combination incorporating high-titanium heavy slag to enhance the flexural crack resistance of concrete beams.

### 4.4. Comparison and Modification of Empirical Formulas for Fracture Characteristics

To further explore the practical applicability of the fracture theory for composite beams, the standard theoretical values of the fracture characteristics were compared with the experimental values. The theoretical formulas were then modified accordingly, with significant deviations.

#### 4.4.1. Comparison of Theoretical and Experimental Values of the Cracking Moment

According to the standards, the cracking moment $M_{cr}$ (when the concrete in the tensile zone cracks) can be calculated using the following formula [40]:

(7) $$f_{c}M_{cr}=\gamma f_{tk}w_{0}$$

where γ is the standard value of the axial compressive strength of concrete; γ is the plasticity influence coefficient of a specimen’s section resistance moment, which is 1.55 for a rectangular section; $w_{0}$ is the elastic section modulus of the converted section’s tensile edge, measured in mm^3^; and $f_{tk}$ is the standard value of the concrete’s tensile strength.

$w_{o}$ is calculated using the following formulas [40]:

(8) $$w_{0}=\frac{I_{0}}{h-y_{0}}$$

(9) $$I_{0}=\frac{by^{3}}{3}+\frac{b\left(h-y_{0}^{3}\right)}{3}+\alpha_{E}A_{S}{\left(h_{0}-y_{0}\right)}^{2}$$

(10) $$y_{0}=\frac{\frac{bh^{2}}{2}+\alpha_{E}A_{s}h_{0}}{bh+\alpha_{E}A_{s}}$$

(11) $$\alpha_{E}=\frac{E_{s}}{E_{c}}$$

where b is the width of the specimen’s cross-section, in millimeters; h is the height of the specimen section, in mm; $y_{0}$ is the distance from the centroid of the converted section to the compression edge; $I_{0}$ is the moment of inertia of the converted section about the centroidal axis; $E_{s}$ is the elastic modulus of the reinforcement; and $E_{c}$ is the elastic modulus of the concrete. The definitions of the other parameters were the same as those listed in Section 4.2.1.

Based on the empirical formulas and experimental results, the experimental cracking-moment values $w_{0te}$ and the theoretical values $w_{0th}$ for the concrete beams were obtained. The cracking-moment error rate E was defined and calculated as follows [36]:

(12) $$E=\frac{\left|w_{0te}{-w}_{0th}\right|}{w_{0te}}\times 100\%$$

**Figure 10:**
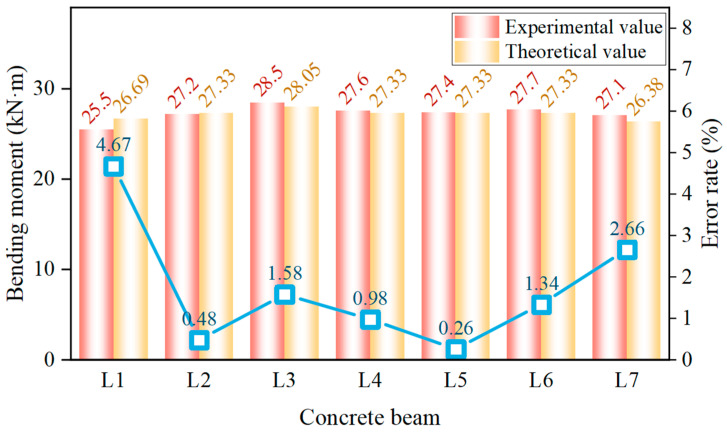
Comparison of experimental and standard theoretical cracking-moment values for composite beams.

The comparison between the experimental cracking-moment values and the standard theoretical values for the seven composite beams after data processing and analysis is shown in Figure 10.

Figure 10 shows that the experimental cracking-moment values for the seven composite beams ranged from 25.5 kN·m to 28.5 kN·m, while the standard theoretical values ranged from 26.28 kN·m to 28.05 kN·m. The beam with the largest error, L1, had an error rate of only 4.67%, demonstrating that the standard calculation formula for the cracking moment of concrete beams was also applicable to high-titanium heavy-slag concrete composite beams.

#### 4.4.2. Comparison of Empirical Formulas for the Maximum Crack Width

To further compare the development of the crack width in the concrete composite beams and discuss the applicability of the crack-width formulas provided by the standards to composite beams, both were analyzed. In accordance with the standards, the maximum crack width $w_{max}$ for concrete beams under bending was calculated as follows [40]:

(13) $$W_{m}=\alpha_{cr}\varphi \frac{\sigma_{s}}{E_{s}}\left(1.9c_{s}+0.08\frac{{\mathrm{d}}_{ep}}{\rho_{te}}\right)$$

(14) $$\varphi =1.1-0.65\frac{f_{tk}}{\rho_{te}\sigma_{s}}$$

(15) $${\mathrm{d}}_{e_{P}}=\frac{\Sigma n_{i}{\mathrm{d}}_{i}^{2}}{\Sigma n_{i}\nu_{i}d_{i}}$$

(16) $$\rho_{te}=\frac{A_{s}}{A_{te}}$$

(17) $$\sigma_{sq}=\frac{M_{sq}}{0.87h_{0}A_{s}}$$

where $\alpha_{cr}$ represents the characteristic coefficient for the member under load, with a value of 1.9 for beams under bending; φ denotes the coefficient of strain non-uniformity for the longitudinal tensile reinforcement in cracks; $\sigma_{sq}$ represents the stress in the longitudinal tensile reinforcement calculated according to a quasi-permanent combination; $c_{s}$ represents the distance from the outer edge of the longitudinal reinforcement to the bottom edge; $A_{te}$ and $A_{s}$ represent the effective cross-sectional area of the tensile concrete zone and the cross-sectional area of the tensile reinforcement; $d_{e_{P}}$ represents the equivalent diameter of the tensile reinforcement; $\rho_{e_{P}}$ represents the reinforcement ratio of the tensile steel bars; and $d_{i},n_{i}$, and $\nu_{i}$ represent the nominal diameter, number, and relative bond characteristic coefficient of the tensile reinforcement, with a value of 1.0 for the ribbed reinforcement. The definitions of the other parameters were the same as those listed in Section 4.2.1.

**Figure 11:**
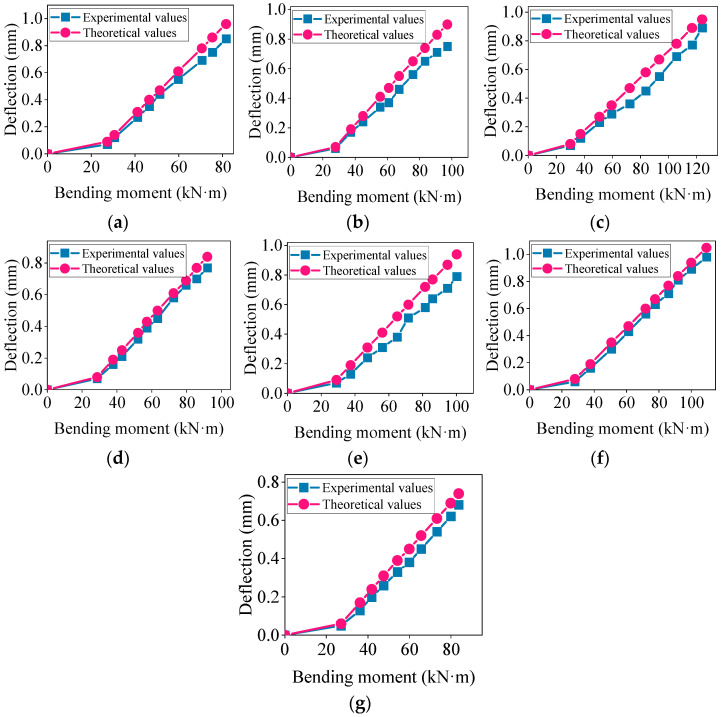
Comparison of experimental and standard theoretical values for maximum crack width: (**a**–**g**) represent the composite beams L1~L7.

The data for the maximum crack width of the concrete beams based on the calculations and experiments are summarized in Figure 11.

From Figure 11, it was observed that beam L6, which was a monolithic beam, showed close agreement between the standard theoretical values and the experimental values for the maximum crack width, validating the accuracy of the experiment. However, for the other composite beams—except for L4, where the standard theoretical value was relatively close to the experimental value—significant errors were noted. Generally, the theoretical values were higher than the actual values and the errors increased with an increase in the bending moment. Our analysis suggested that due to structural differences, the load-carrying mechanisms of the composite beams differed from the monolithic beams, necessitating some adjustments to the empirical formulas provided by the standards to better suit composite beams.

#### 4.4.3. Correction and Re-Comparison of Empirical Formulas for the Maximum Crack Width

To improve the accuracy of the calculations of the maximum crack width in composite beams, a linear regression was performed between the theoretical values and experimental results, introducing a correction factor θ. The linear regression equations were [40]:

(18) θ=1.05−1.04h

(19) $$W_{m}={\theta \alpha }_{cr}\varphi \frac{\sigma_{s}}{E_{s}}\left(1.9c_{s}+0.08\frac{d_{ep}}{\rho_{te}}\right)$$

where h represents the prefabricated height of the composite beam, in meters. The definitions of other parameters were the same as those listed in Section 4.2.1.

**Figure 12:**
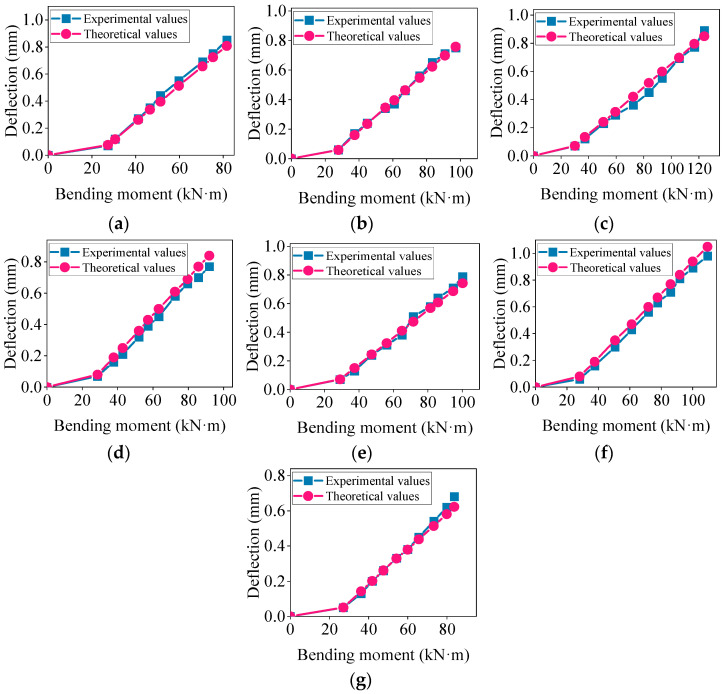
Comparison of experimental and corrected theoretical values for maximum crack width: (**a**–**g**) represent the composite beams L1~L7.

The results of the corrected maximum crack widths compared with the experimental results are shown in Figure 12.

Figure 12 illustrates that by introducing the correction factor θ, and based on the prefabricated height of the composite beam, the theoretical values of the maximum crack width for six concrete composite beams aligned more closely with the experimental values. Similar to previous analyses, the error rate E was defined to further quantify the deviation of the corrected theoretical values and was calculated as follows [36]:

(20) $$E=\frac{\left|\overset{n}{\underset{i=1}{\sum }}{W_{m}}_{tei}–{W_{m}}_{thi}\right|}{\overset{n}{\underset{i=1}{\sum }}{W_{m}}_{tei}}\times 100\%$$

where E represents the calculation error, ${W_{m}}_{tei}$ denotes the maximum crack width of the concrete beam obtained from the experimental tests, and ${W_{m}}_{thi}$ represents the maximum crack width of the concrete beam calculated using the formula.

**Figure 13:**
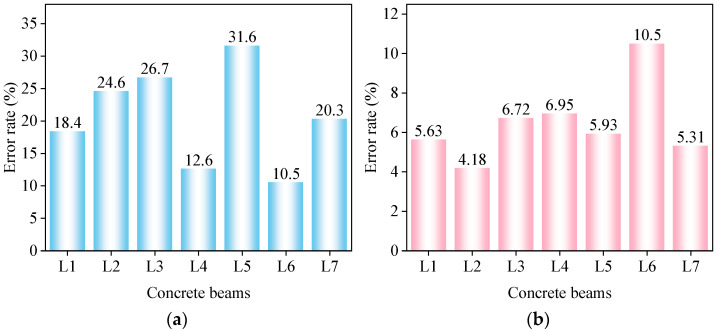
Error comparison of crack width: (**a**) standard theoretical values; (**b**) modified theoretical values.

The error comparison between the corrected theoretical values and the standard theoretical values for the maximum crack width of the concrete composite beams according to the calculations is shown in Figure 13.

Figure 13 shows that before the modification, the standard theoretical formula for the maximum crack width for concrete beams had significant deviations, with the largest error for beam L5 at 31.6% and the smallest for beam L4 at 12.6%. Our analysis suggested that the differing heights of the prefabricated sections (250 mm for L5 and 150 mm for L4) contributed to the discrepancies between the theoretical and actual values.

After introducing the modified formulas, the theoretical calculations for all seven composite beams aligned more closely with the experimental values, with errors reduced to within 10%. This indicated that the modified formulas were effective, significantly improving the accuracy of the theoretical formulas for the maximum crack width of flexural composite beams, thereby enhancing the safety and reliability of composite-beam design.

## 5. Conclusions

This study conducted static bending load tests on high-titanium heavy-slag concrete composite beams to investigate the effects of parameters such as the longitudinal reinforcement ratio, height of the prefabricated layer, and material structure combinations on the deflection development and fracture characteristics of these beams. Empirical formulas from the standards for deflection development and crack propagation were compared with experimental values. Theoretical formulas with significant deviations from the actual values were modified and re-compared. Based on the research, the main conclusions were as follows:

(1) The variables of the composite beams, such as the composite height, reinforcement ratio, and material structure combination, significantly impacted the deflection and cracking of composite beams. High-titanium heavy slag and fly-ash ceramsite enhanced the composite beam’s deformation resistance. To reduce the deflection of the bending members and mitigate crack development, it is recommended that the reinforcement ratio is appropriately increases and high-titanium heavy-slag composite-beam material structures are used.
(2) A comparison of the theoretical and experimental values of the cracking moment revealed minimal deviations, indicating that the empirical formula for the cracking moment of ordinary concrete beams was equally applicable to high-titanium heavy-slag concrete composite beams.
(3) Significant discrepancies existed between the theoretical and experimental values for the deflection development and maximum crack width of concrete beams. Linear fitting and the introduction of a coefficient related to the prefabricated height of the composite beam corrected the formula, reducing the deflection development deviation to within 5% and the maximum crack-width deviation to within 10%. This adjustment aligned the theoretical design values more closely with the actual values, providing theoretical support to the use of composite beams.

## Figures and Tables

**Table 1:** Main performance indicators of cement.

Compressive Strength (MPa)		Flexural Strength (MPa)		Setting Time (Min)	
3 d	28 d	3 d	28 d	Initial Setting Time	Final Setting Time
27.8	46.6	4.8	7.2	175	250

**Table 2:** Sieve analysis of high-titanium heavy-slag crushed stone.

Aggregate Particle Size (mm)	26	19	11.08	5.45	2.28
Fractional sieve retention (%)	2.7	2.3	35	46	7
Cumulative sieve retention (%)	2.7	5	40	86	93

**Table 3:** Mechanical properties’ test results for steel bars.

Diameter (mm)	Yield Strength (N/mm^2^)	Average Yield Strength (N/mm^2^)	Ultimate Strength (N/mm^2^)	Average Ultimate Strength (N/mm^2^)	Modulus of Elasticity (N/mm^2^)
16	473.84	471.06	686.88	650.66	2.05 × 10^5^
16	468.28		689.15		
18	480.23	479.06	648.86	667.47	2.07 × 10^5^
18	477.88		652.45		
20	501.28	499.89	666.53	688.12	2.09 × 10^5^
20	498.49		668.41		

**Table 4:** Design of high-titanium heavy-slag concrete material mix proportions.

Water (kg/m^3^)	Cement (kg/m^3^)	High-Titanium Heavy-Slag Crushed Stone (kg/m^3^)	High-Titanium Heavy-Slag Sand (kg/m^3^)	Fly-Ash Aggregate (kg/m^3^)	Fly Ash (kg/m^3^)	Silica Fume (kg/m^3^)	Water-Reducing Agent (kg/m^3^)
210	312.21	588.77	452.90	252.33	14.19	28.38	1.56

**Table 5:** Design of ordinary concrete material mix proportions.

Water (kg/m^3^)	Cement (kg/m^3^)	Limestone Coarse Aggregate (kg/m^3^)	Limestone Fine Aggregate (kg/m^3^)	Fly Ash (kg/m^3^)	Silica Fume (kg/m^3^)	Water-Reducing Agent (kg/m^3^)
210	312.21	731.39	562.61	14.19	28.38	1.56

**Table 6:** Design of material–structure combinations for high-titanium heavy-slag concrete.

Specimen	L1	L2	L3	L4	L5	L6	L7
Composite Part	HTC	HTC	HTC	HTC	HTC	HTC	OPC
+	200 mm	200 mm	200 mm	150 mm	250 mm		200 mm
Prefabricated Part	HTC	HTC	HTC	HTC	HTC		OPC
Section Dimensions (mm)	200 × 400	200 × 400	200 × 400	200 × 400	200 × 400	200 × 400	200 × 400
Cover Thickness (mm)	25	25	25	25	25	25	25
Erecting Bars	2C10	2C10	2C10	2C10	2C10	2C10	2C10
Tensile Bars	2C16	2C18	2C20	2C18	2C18	2C18	2C18
Stirrups	C8@10	C8@10	C8@10	C8@10	C8@10	C8@10	C8@10

## Data Availability

The original contributions presented in the study are included in the article, further inquiries can be directed to the corresponding author.
